# Adverse Outcome Following Mild Traumatic Brain Injury Is Associated with Microstructure Alterations at the Gray and White Matter Boundary

**DOI:** 10.3390/jcm12165415

**Published:** 2023-08-21

**Authors:** Lara Pankatz, Philine Rojczyk, Johanna Seitz-Holland, Sylvain Bouix, Leonard B. Jung, Tim L. T. Wiegand, Elena M. Bonke, Nico Sollmann, Elisabeth Kaufmann, Holly Carrington, Twishi Puri, Yogesh Rathi, Michael J. Coleman, Ofer Pasternak, Mark S. George, Thomas W. McAllister, Ross Zafonte, Murray B. Stein, Christine E. Marx, Martha E. Shenton, Inga K. Koerte

**Affiliations:** 1 Psychiatry Neuroimaging Laboratory, Department of Psychiatry, Brigham and Women’s Hospital, Harvard Medical School, Somerville, MA 02145, USA; lara.pankatz@med.uni-muenchen.de (L.P.); philine.rojczyk@med.uni-muenchen.de (P.R.); jseitz@bwh.harvard.edu (J.S.-H.); sylvain.bouix@etsmtl.ca (S.B.); leonard.jung@med.uni-muenchen.de (L.B.J.); tim.wiegand@med.uni-muenchen.de (T.L.T.W.); elena.bonke@med.uni-muenchen.de (E.M.B.); nico.sollmann@tum.de (N.S.); elisabeth.kaufmann@med.uni-muenchen.de (E.K.); holly.carrington@mountsinai.org (H.C.); tpuri1@bwh.harvard.edu (T.P.); yogesh@bwh.harvard.edu (Y.R.); mjc@bwh.harvard.edu (M.J.C.); ofer@bwh.harvard.edu (O.P.); shenton@bwh.harvard.edu (M.E.S.); 2 cBRAIN, Department of Child and Adolescent Psychiatry, Psychosomatic and Psychotherapy, Ludwig-Maximilians-Universität, 80336 Munich, Germany; 3 Department of Psychiatry, Massachusetts General Hospital, Harvard Medical School, Boston, MA 02114, USA; 4 Département de génie logiciel et TI, École de Technologie Supérieure, Université du Québec, Montreal, QC H3C 1K3, Canada; 5 Graduate School of Systemic Neuroscience, Ludwig-Maximilians-Universität, 82152 Planegg, Germany; 6 Department of Diagnostic and Interventional Radiology, University Hospital Ulm, 89081 Ulm, Germany; 7 Department of Diagnostic and Interventional Neuroradiology, School of Medicine, Klinikum rechts der Isar, Technical University of Munich, 81675 Munich, Germany; 8 TUM-Neuroimaging Center, Klinikum rechts der Isar, Technical University of Munich, 81675 Munich, Germany; 9 Department of Neurology, University Hospital, LMU, 81377 Munich, Germany; 10 Brain Injury Research Center of Mount Sinai, Icahn School of Medicine at Mount Sinai, New York, NY 10029, USA; 11 Department of Radiology, Brigham and Women’s Hospital, Harvard Medical School, Boston, MA 02115, USA; 12 Psychiatry Department, Medical University of South Carolina, Charleston, SC 29425, USA; georgem@musc.edu; 13 Ralph H. Johnson VA Medical Center, Charleston, SC 29401, USA; 14 Department of Psychiatry, Indiana University School of Medicine, Indianapolis, IN 46202, USA; twmcalli@iupui.edu; 15 Department of Physical Medicine and Rehabilitation, Spaulding Rehabilitation Hospital, Harvard Medical School, Charlestown, MA 02129, USA; rzafonte@partners.org; 16 Department of Physical Medicine and Rehabilitation, Brigham and Women’s Hospital, Harvard Medical School, Boston, MA 02115, USA; 17 Department of Psychiatry, University of California San Diego, La Jolla, CA 92093, USA; mstein@ucsd.edu; 18 School of Public Health, University of California San Diego, La Jolla, CA 92093, USA; 19 Psychiatry Service, VA San Diego Healthcare System, San Diego, CA 92161, USA; 20 VA Mid-Atlantic Mental Illness Research and Clinical Center (MIRECC) and Durham VA Medical Center, Durham, NC 27705, USA; christine.marx@duke.edu; 21 Department of Psychiatry and Behavior Sciences, Duke University School of Medicine, Durham, NC 27710, USA

**Keywords:** diffusion tensor imaging, mild traumatic brain injury, fractional anisotropy, magnetic resonance imaging, post-concussion symptoms, cognitive impairment

## Abstract

The gray matter/white matter (GM/WM) boundary of the brain is vulnerable to shear strain associated with mild traumatic brain injury (mTBI). It is, however, unknown whether GM/WM microstructure is associated with long-term outcomes following mTBI. The diffusion and structural MRI data of 278 participants between 18 and 65 years of age with and without military background from the Department of Defense INTRuST study were analyzed. Fractional anisotropy (FA) was extracted at the GM/WM boundary across the brain and for each lobe. Additionally, two conventional analytic approaches were used: whole-brain deep WM FA (TBSS) and whole-brain cortical thickness (FreeSurfer). ANCOVAs were applied to assess differences between the mTBI cohort (*n* = 147) and the comparison cohort (*n* = 131). Associations between imaging features and post-concussive symptom severity, and functional and cognitive impairment were investigated using partial correlations while controlling for mental health comorbidities that are particularly common among military cohorts and were present in both the mTBI and comparison group. Findings revealed significantly lower whole-brain and lobe-specific GM/WM boundary FA (*p* < 0.011), and deep WM FA (*p* = 0.001) in the mTBI cohort. Whole-brain and lobe-specific GM/WM boundary FA was significantly negatively correlated with post-concussive symptoms (*p* < 0.039), functional (*p* < 0.016), and cognitive impairment (*p* < 0.049). Deep WM FA was associated with functional impairment (*p* = 0.002). Finally, no significant difference was observed in cortical thickness, nor between cortical thickness and outcome (*p* > 0.05). Findings from this study suggest that microstructural alterations at the GM/WM boundary may be sensitive markers of adverse long-term outcomes following mTBI.

## 1. Introduction

Traumatic brain injury (TBI) is a leading cause of disability and is particularly common among military service members [1,2]. About 90% of TBI cases are categorized as mild TBI (mTBI) and affect as many as ~42 million people annually worldwide [3,4]. About a third of individuals with mTBI will go on to develop long-term symptoms, also referred to as persistent post-concussive symptoms [5]. Nonetheless, and despite the high prevalence of mTBI and the large number of individuals with adverse long-term outcomes, the underlying pathophysiology is still not fully understood.

The current understanding is that during a head impact, rotational and linear forces act on the skull, leading to brain tissue deformation and, thereby the stretching and shearing of axons [6]. The latter may result in microinjury of axons, also known as traumatic axonal injury (TAI), which is one of the most common injuries associated with mTBI [7]. The gray matter/white matter (GM/WM) boundary is particularly susceptible to TAI, given that WM is more rigid and viscous than GM and reacts less rapidly to mechanical strain, potentially leading to the shearing of the two types of tissues juxtaposed to one another [8,9]. Indeed, recent computer simulations suggest that the GM/WM boundary is where acceleration and shear forces are at their highest levels [10]. Moreover, microbleeds, which constitute a prevalent pathophysiologic feature of brain tissue strain are commonly found along the GM/WM boundary [11]. Of note, WM microinjuries to the brain tissue have previously been associated with the development of long-term post-concussive symptoms [12]. These symptoms may involve cognitive and functional disability including compromised processing speed, executive functioning, and problems with participation in social activities, work, and family life [13].

Strikingly, microinjuries to WM often remain undetected by conventional imaging modalities (e.g., computed tomography (CT)) and thus their detection requires the use of more sensitive advanced imaging techniques. Importantly, diffusion magnetic resonance imaging (dMRI) can both detect and quantify even subtle alterations in brain structure. Specifically, dMRI measures the magnitude and orientation of water molecule diffusion in brain tissue, expressed as fractional anisotropy (FA) [14]. 

Previous studies investigating individuals with a history of mTBI have focused on brain alterations of either the most central part of WM (“deep WM”) [15,16] or GM [17,18]. Interestingly, these studies revealed variable findings, with some reporting increases or decreases in FA in the chronic phase following mTBI [15], while others did not observe differences in FA between mTBI individuals and a comparison group [16]. Similarly, when assessing GM structure following mTBI, some studies report alterations in GM cortical thickness [17], while other studies do not reveal any differences in GM compared to controls [18] in the acute and sub-acute stages after mTBI. Conflicting results have further been reported in the chronic stages, where some studies describe thinning of the cortex [19], while others report thickening in the same GM regions [20]. In fact, time since injury is an important aspect when considering brain structural alterations following mTBI. Some studies have suggested that even in the case that rehabilitative processes lead to presumed recovery after a couple of weeks or months, degenerative processes may commence later in life and may be linked to disadvantageous outcomes, such as accelerated aging and declining cognitive functioning [21,22]. It is possible that previous studies did not observe alterations in brain microstructure following mTBI because they failed to assess what may constitute the most vulnerable region of injury, and that is the GM/WM boundary. This may also explain why some individuals with mTBI exhibit post-concussive symptoms despite any visible signs of tissue alteration in either the deep WM [23] or the cortex [24]. 

The aim of this study is to characterize the microstructure at the GM/WM boundary in participants of the Department of Defense Injury and Traumatic Stress (INTRuST) study with a history of mTBI against a comparison group while adjusting for common confounders of brain structure such as age, gender, post-traumatic stress disorder (PTSD), depressive symptoms, and alcohol use. In addition, we investigate whether alterations in GM/WM boundary diffusion properties are associated with clinical outcome measures following mTBI, including post-concussive symptom severity, cognitive functioning, and functional impairment. Finally, since this is the first investigation focusing on the GM/WM boundary in this population, we also apply more commonly used approaches to investigate brain structure following mTBI (i.e., WM diffusion of the deep WM and cortical thickness).

## 2. Materials and Methods

### 2.1. Study Design and Participants

Participants of the Injury and Traumatic Stress (INTRuST) Clinical Consortium (Department of Defense, W81XWH-08-2-0159, intrust.sdsc.edu, accessed on 6 January 2022) were enrolled from 10 sites across the United States between 2008 and 2013. Institutional review board study approval was obtained from all participating sites and the study was conducted in conformity with the Declaration of Helsinki. All study participants provided written informed consent prior to enrolment. 

Participants included English-speaking males or females between the ages of 18 and 70 years of age. Participants were excluded from the study if they had acquired English as a second language after the age of 5, had a history of a learning disability, a TBI history that resulted in a hospital stay and/or abnormal imaging findings, a history of moderate to severe TBI, a diagnosis of Bipolar I, psychotic, delirious, or dementia-related disorders, uncontrolled chronic disease, uncontrolled hypertension, or were taking more than one antihypertensive medication, used oral or intramuscular steroids within the past four months, or were currently taking drugs or any medication affecting brain function (other than psychotropic medication). Individuals with a history of psychotropic drug, alcohol, or substance use were permitted to participate in the study if they had been in remission for the last 30 days prior to data collection. Additional exclusion criteria were general MRI contraindications, disorders of the central nervous system, or pregnancy/lactating. 

Out of the 771 enrolled participants, 373 completed neuropsychological and MRI assessments. After the evaluation of MRI data quality, 95 cases were excluded due to severe motion artifacts (*n* = 14), harmonization issues (*n* = 44), or missing demographic data (*n* = 37) leaving a total of 278 participants with available structural and dMRI data. The enrolled participants did not differ significantly from those excluded in any demographic variable (*p* > 0.05). These participants were classified into participants with a history of mTBI (*n* = 147) and a comparison group without mTBI (*n* = 131) (Table 1). Participants with psychiatric comorbidities (other than severe mental disorders such as schizophrenia and bipolar disorder) were not excluded from either group given that we aimed for a most accurate resemblance of the larger population, and psychiatric symptoms are common in the military population and those with mTBI.

### 2.2. Diagnostic and Clinical Assessments

#### 2.2.1. Assessment of mTBI

Mild TBI history was assessed using the self-report INTRuST mTBI Screening Instrument which closely follows the American Congress of Rehabilitation Medicine’s diagnostic guidelines [25] and has been used in previous publications of the INTRuST Clinical Consortium [26]. MTBI was diagnosed if a head injury led to any of the following: alteration or loss of consciousness and/or posttraumatic anterograde/retrograde amnesia.

#### 2.2.2. Assessment of Post-Concussion Symptom Severity

The Rivermead Post-Concussion Symptoms Questionnaire (RPQ13) [27] is an established scale for assessing chronic persistent post-concussive symptoms with strong internal validity, test–retest reliability, and inter-rater reliability [28]. Higher scores on the RPQ13 indicate greater post-concussive symptom severity.

#### 2.2.3. Assessment of Functional Impairment

To assess functional impairment, the Sheehan Disability Scale (SDS) was employed [29]. The SDS is a self-report questionnaire that includes three items that assess functional impairment in daily work, social, and family life (e.g., “symptoms have disrupted family/home responsibilities”). The scale is rated from 0 (“not at all”) to 10 (“extreme”). This scale was used to assess functional impairment in daily work, social, and family life. Higher scores on the SDS indicate greater functional impairment.

#### 2.2.4. Assessment of Cognitive Functioning 

The Trail Making Test (TMT) [30] is a highly efficient tool to assess cognitive functioning, such as processing speed (TMT-A) and executive functioning, including mental flexibility and task switching, attention, and visual tracking (TMT-B). TMT-A assesses how fast a participant can connect 25 numbers in ascending order (i.e., 1-2-3 etc.) that are randomly arranged on a sheet of paper. TMT-B requires the participant to alternate between connecting letters and numbers in numerical and alphabetical order, respectively (i.e., 1-A-2-B etc.). For both TMT-A and B, the total time to completion is assessed. The TMT is sensitive in discriminating between individuals with brain alterations and healthy controls [31]. 

#### 2.2.5. Assessment of Psychiatric Comorbidities 

The presence and severity of PTSD symptoms were assessed using the PTSD Checklist-Civilian Version (PCL-C) [32], a 17-item self-report questionnaire corresponding to the Diagnostic and Statistical Manual of Mental Disorders IV (DSM-IV) diagnostic criteria for PTSD. Items (e.g., “repeated, disturbing memories, thoughts, or images of a stressful experience from the past?”) are rated on a scale from 1 “not at all bothersome” to 5 “extremely bothersome,” and summed into a total symptom severity score.

The severity of depressive symptoms was assessed using the 9-Item Patient Health Questionnaire 9 (PHQ-9), with items rated from 0 “not at all” to 3 “nearly every day” [33]. A total symptom severity score was computed by summing all items.

The Alcohol Use Disorders Identification Test (AUDIT-10) was used to assess alcohol consumption [34]. The AUDIT-10 consists of 10 items that cover the quantity of alcohol consumed, level of dependency, and harmful consequences for self and others. Items (e.g., “How many drinks containing alcohol do you have on a typical day when you are drinking?”) are scored on a scale from 0 to 4 with varying response options depending on the question. A total alcohol use severity score was computed by summing all items. Higher scores correspond to greater alcohol use severity. 

### 2.3. MRI Acquisition and Image Processing

#### 2.3.1. Image Acquisition

Structural MRI sequences and dMRI sequences were acquired on 3-Tesla scanners (Tim Trio, Siemens Healthineers, Erlangen, Germany; GE 750, GE Healthcare, Chicago, IL, USA, or Achieva, Philips Healthcare, Best, The Netherlands) across six out of ten INTRuST study sites (for details for each MRI system, see Table 2).

#### 2.3.2. Image Pre-Processing

Pre-processing of the structural and dMRI images was conducted according to the in-house pipeline of the Psychiatry Neuroimaging Laboratory, Brigham and Women’s Hospital, Harvard Medical School (https://github.com/pnlbwh/pnlNipype, accessed on 10 December 2021). This included axis alignment, centering, and motion correction of all images. For the dMRI images, eddy current correction was applied. The image quality was visually inspected and semi-automatically examined for artifacts (e.g., motion artifacts) using 3D Slicer (version 4.5, Surgical Planning Laboratory, Brigham and Women’s Hospital, Boston, MA, USA; http://www.slicer.org, accessed on 10 December 2021) [35]. Structural and diffusion masks covering the entire brain were constructed and manually corrected in 3D Slicer where necessary (e.g., in case of incomplete coverage of the brain) by a trained rater.

#### 2.3.3. Structural Image Processing

To accurately delineate the GM/WM boundary, FreeSurfer was used (version 5.3, Laboratory for Computational Neuroimaging, Boston, MA, USA; https://surfer.nmr.mgh.harvard.edu, accessed 15 January 2022) [36]. Processing of the structural data included the removal of non-brain tissue, automated Talairach transformation, grayscale intensity normalization, correction for any inhomogeneities in the magnetic field, automated topology correction, and surface deformation correction referring to intensity gradients. Subsequent steps included surface inflation, registration to a common spherical atlas, and parcellation of the cortex into 150 regions of interest (ROIs, 75 per hemisphere) according to the Destrieux brain atlas [37]. This resulted in a 3D reconstruction of the GM/WM boundary, which was visually quality checked to ensure anatomical accuracy.

To account for alterations in the GM, cortical thickness was calculated as the distance between the GM/WM boundary (i.e., white surface) and the GM/cerebrospinal fluid (CSF) boundary (i.e., pial surface) [38]. Measurements of cortical thickness were smoothed using a standard Gaussian kernel to enhance contrast.

#### 2.3.4. Diffusion-Weighted Image Processing

Given the multi-site nature of the INTRuST project and the associated scanner variability, an established single-shell harmonization algorithm (https://github.com/pnlbwh/dMRIharmonization, accessed 10 January 2022) was used on the b = 900 dMRI data to account for scanner differences, while maintaining within-site inter-subject variability [39]. Using a least-squares fit model to derive diffusion tensors from the preprocessed diffusion-weighted images, voxel-based FA maps were produced. FA is a diffusion metric that ranges between 0 (*isotropic*, unrestricted diffusion) and 1 (*anisotropic*, restricted diffusion) and expresses the magnitude and direction of water molecule diffusion in the tissue [40]. Additionally, free-water (FW) imaging was used to estimate and eliminate the relative contribution of extracellular FW (e.g., due to CSF, edema, or atrophy) [41]. After accounting for FW, corrected FA maps were extracted.

Deep WM was examined using tract-based spatial statistics (TBSS; https://github.com/pnlbwh/TBSS, accessed 26 January 2022) [42], according to the Enhancing Neuro Imaging Genetics by Meta-Analysis Diffusion Tensor Imaging (ENIGMA-DTI) working group’s protocol (http://enigma.ini.usc.edu/ongoing/dti-working-group/, accessed 26 January 2022). The FA maps generated were co-registered onto the ENIGMA-DTI template and subsequently projected onto the ENIGMA-DTI skeleton [43]. A skeletonized FA map was produced, depicting the core of each participant’s WM fiber pathways. Last, average FW-corrected FA values were extracted from each participant’s FA skeleton.

#### 2.3.5. Registration and Extraction of Diffusion Metrics at the GM/WM Boundary

**Figure 1:**
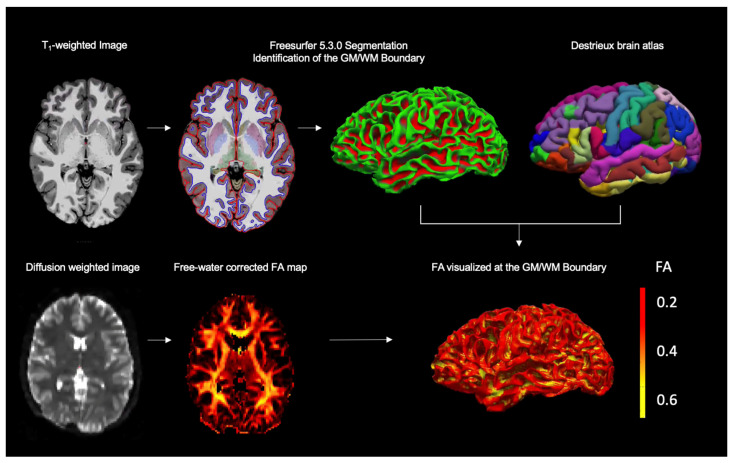
**Image Processing.** Structural MRI sequences were segmented into GM and WM using FreeSurfer 5.3.0, to identify the GM/WM boundary (in blue). Diffusion MRI (dMRI) images were fitted with the free-water (FW) map to create a voxel-wise map of FA corrected for FW. Each participant’s FW-corrected dMRI data were registered onto the respective FreeSurfer segmentation. All images were overlayed with the Destrieux brain atlas to identify and extract FA values along the GM/WM intersection in different regions of the brain. *Note.* GM, gray matter; WM, white matter; FA, fractional anisotropy _Tissue_; Images are displayed using Freeview. The FW-corrected FA map is shown in color scheme ‘Heat’ for better visualization.

To estimate FA values along the GM/WM boundary, we registered the b = 0 map from the dMRI scan to the Freesurfer subject’s space using boundary-based registration [10,44] (FS command: bbregister, option –t2 to indicate the b = 0 contrast is similar to a T2 weighted image). The resulting transformation was then used to co-register and map the subject’s FA image (in original dMRI space) to the Freesurfer “white” left and right hemisphere surfaces (FS command: mri_vol2surf) (Figure 1). We then extracted FA values for the GM/WM boundary across the brain (whole-brain), as well as for each of the major brain lobes, frontal, parietal, temporal, and occipital lobe according to the Destrieux brain atlas [37]. When merging ROIs, the average FA of the merged region was computed via a weighted sum of the mean FA of the individual ROIs to account for area differences between individual ROIs. 

### 2.4. Statistical Analysis 

SPSS software (version 25.0; IBM Statistics for Mac, Armonk, NY, USA) was used for all statistical analyses. Age, gender, PTSD symptoms (PCL-C), depressive symptoms (PHQ-9), and alcohol use (AUDIT-10) were included as covariates in all models to account for potentially confounding effects on brain structure. Significance values were corrected for multiple comparisons according to the Benjamini–Hochberg method (false discovery rate (FDR)) [45]. *p*-values were adjusted for seven analyses referring to seven outcome measures (whole-brain, frontal, parietal, temporal, and occipital lobe GM/WM boundary FA, deep WM FA, and cortical thickness). An FDR-corrected *p*-value of 0.05 was set to indicate statistical significance. 

#### 2.4.1. Group Differences in GM/WM Boundary Diffusion, Deep WM Diffusion, and Cortical Thickness

We conducted seven analyses of covariance (ANCOVAs) to assess differences in whole-brain, frontal, parietal, temporal, and occipital lobe GM/WM boundary FA, deep WM FA, and cortical thickness (respective dependent variables) between groups of participants with mTBI compared to the comparison group without mTBI (independent variable). 

#### 2.4.2. Correlation between GM/WM Boundary Diffusion and Post-Concussive Symptoms, Functional Impairment, and Cognitive Functioning

To determine whether GM/WM boundary diffusion serves as an indicator of long-term outcome after mTBI, we assessed correlations between whole-brain, frontal, parietal, temporal, and occipital lobe GM/WM boundary FA, deep WM FA, cortical thickness, and chronic post-concussive symptoms (RPQ13), functional impairment (in work, social, and family life (SDS)), and cognitive functioning (processing speed (TMT-A) and executive functioning (TMT-B)). Nonparametric partial correlations were used due to non-normal distributions of the outcome variables.

The data presented in this study is available upon request from the corresponding author.

## 3. Results

The sample characteristics and of the study sample are presented in Table 1**,** together with summary statistics and analytical results. The age and racial distribution between the groups did not differ (*p* > 0.05). However, there was an unequal gender distribution between the two groups (*p* < 0.001). ANCOVA further revealed greater PTSD and depressive symptoms in the mTBI group (*p* < 0.001), as well as higher alcohol consumption (*p* = 0.011). Furthermore, the mTBI group displayed greater chronic post-concussive symptoms (*p* = < 0.001) and significantly greater functional impairment (*p* = < 0.001). The mTBI group showed worse processing speed than the comparison group (*p* = 0.014), however, there was no significant difference between the groups for executive functioning (*p* = 0.348).

### 3.1. Group Differences in GM/WM Boundary Diffusion, Deep WM Diffusion, and Cortical Thickness

**Figure 2:**
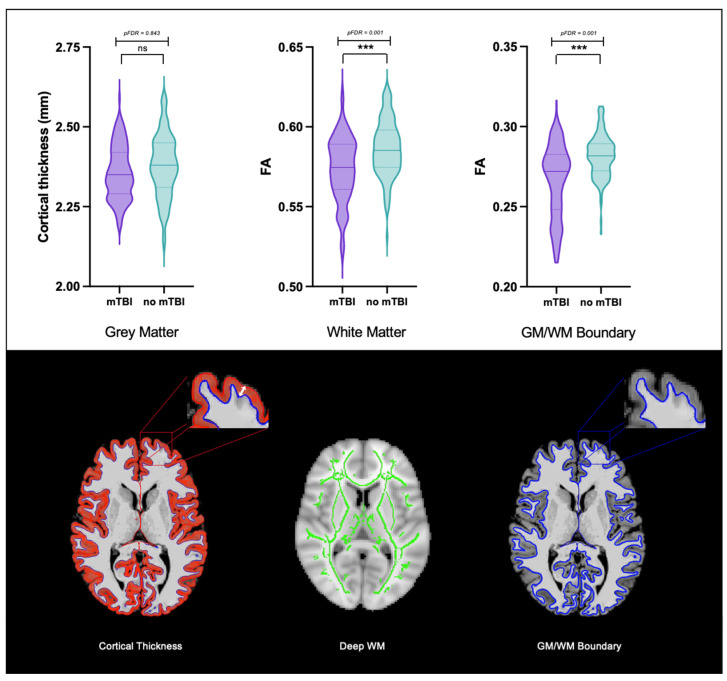
**Group differences between participants with mTBI and participants without mTBI.** ANCOVA revealed significantly lower whole-brain GM/WM boundary FA (pFDR = 0.001), and deep WM FA (pFDR = 0.001) in mTBI subjects compared to participants without mTBI. No significant differences were observed between groups in cortical thickness (pFDR = 0.843). The area of the brain where the imaging measure was acquired is displayed beneath for better visualization. *Note*. mTBI, Mild traumatic brain injury; FA, Fractional Anisotropy; ns = not significant; *** = *p* < 0.001.

The ANCOVAs revealed significant lower whole-brain (*p* < 0.001, pFDR = 0.001), frontal (*p* < 0.001, pFDR = 0.001), parietal (*p* = 0.003, pFDR = 0.004), temporal (*p* < 0.001, pFDR = 0.001), and occipital lobe (*p* = 0.010, pFDR = 0.011) GM/WM boundary FA and deep WM FA (*p* < 0.001, pFDR = 0.001) in the mTBI compared to the no mTBI group. However, no significant differences were found for cortical thickness (*p* = 0.843, pFDR = 0.843) between the mTBI group and the no mTBI group. (Table 1, Figure 2). 

### 3.2. Correlation between GM/WM Boundary Diffusion and Post-Concussive Symptoms, Functional Impairment, and Cognitive Functioning

Having more post-concussive symptoms (RPQ13) was significantly negatively correlated with lower whole-brain (r = −0.19, *p* = 0.017, pFDR = 0.039), frontal (r = −0.21, *p* = 0.007, pFDR = 0.024), and parietal (r = −0.21, *p* = 0.006, pFDR = 0.024) GM/WM boundary FA_T_, but not with temporal (r = −0.16, *p* = 0.043, pFDR = 0.075) and occipital GM/WM boundary FA_T_ (r = 0.06, *p* = 0.404, pFDR = 0.404), deep WM FA_T_ (r = −0.12, *p* = 0.117, pFDR = 0.163), or cortical thickness (r = 0.10, *p* = 0.201, pFDR = 0.234).

The analyses further revealed significant associations between functional impairment (SDS) and lower whole-brain (r = −0.23, *p* < 0.001, pFDR = 0.002), frontal (r = −0.19, *p* = 0.001, pFDR = 0.002), parietal (r = −0.17, *p* = 0.004, pFDR = 0.006), temporal (r = −0.25, *p* = < 0.001, pFDR = 0.002), and occipital (r = −0.15, *p* = 0.014, pFDR = 0.016) GM/WM boundary FA_T_ as well as deep WM FA_T_ (r = 0.25, *p* < 0.001, pFDR = 0.002), but not for cortical thickness (r = −0.05, *p* = 0.413, pFDR = 0.413).

Moreover, significant associations were found between slower processing speed (TMT A) and lower whole-brain (r = −0.16, *p* = 0.009, pFDR = 0.031), frontal (r = −0.17, *p* = 0.006, pFDR = 0.031), and temporal (r = −0.14, *p* = 0.021, pFDR = 0.049) GM/WM boundary FA_T_, but not for lower processing speed with parietal (r = −0.11, *p* = 0.072, pFDR = 0.101) or occipital GM/WM boundary FA_T_ (r = −0.05, *p* = 0.448, pFDR = 0.448), deep WM FA_T_ (r = −0.12, *p* = 0.042, pFDR = 0.073), or cortical thickness (r = −0.05, *p* = 0.441, pFDR = 0.448).

**Figure 3:**
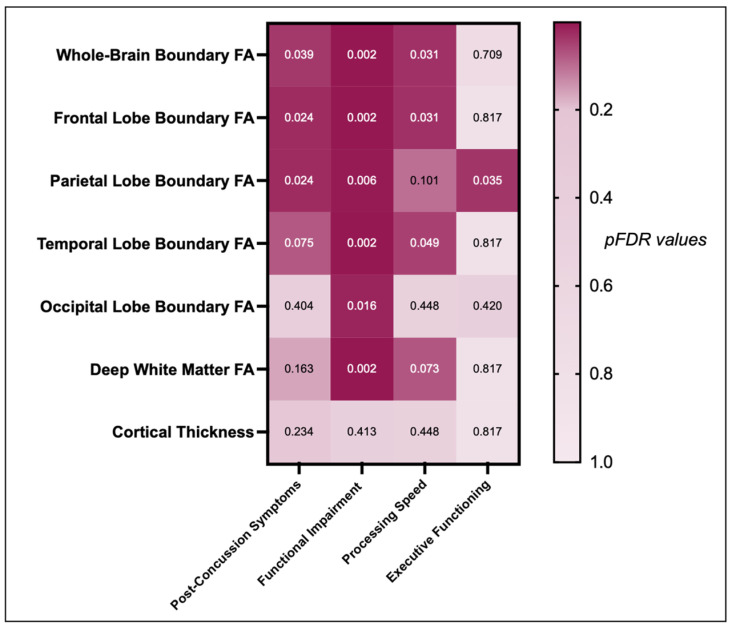
Association between GM/WM boundary diffusion, deep white matter, cortical thickness, and long-term outcomes (symptoms, functional impairment, processing speed, executive functioning), displayed by a heatmap of *p*FDR-values. Dark red boxes indicate a stronger relationship between imaging and outcome measures, whereas brighter boxes indicate a weak relationship between imaging and outcome measures. *Note.* FA, Fractional anisotropy.

**Figure 4:**
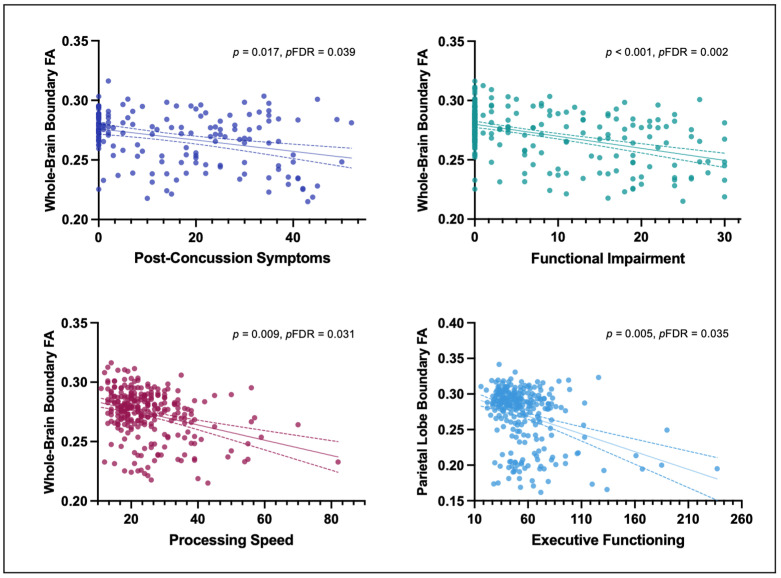
Associations between GM/WM boundary diffusion, post-concussive symptoms, functional impairments, and cognitive functioning. In all analyses, GM/WM boundary diffusion was negatively correlated with the outcome measure, suggesting an association between lower GM/WM diffusion and poorer long-term outcome. *Note.* FA, Fractional anisotropy; Post-Concussion Symptoms (RPQ13 Score); Functional Impairment (SDS Score); Processing Speed (TMT-A time in seconds); Executive Functioning (TMT-B time in seconds).

Last, significant associations were found between poorer executive functioning (TMT B) and lower parietal GM/WM boundary FA_T_ (r = −0.17, *p* = 0.005, pFDR = 0.035), but not whole-brain (r = −0.06, *p* = 0.304, pFDR = 0.709), frontal (r = −0.02, *p* = 0.716, pFDR = 0.817), temporal (r = −0.03, *p* = 0.663, pFDR = 0.817), or occipital GM/WM boundary FA_T_ (r = −0.10, *p* = 0.120, pFDR = 0.420), deep WM FA_T_ (r = −0.01, *p* = 0.817, pFDR = 0.817), or cortical thickness (r = −0.04, *p* = 0.477, pFDR = 0.817) (Figure 3 and Figure 4).

## 4. Discussion

This study found that the group with a history of mTBI (mean 7.57 ± 9.54 years after mTBI) had significantly lower whole-brain and lobe-specific GM/WM boundary FA, as well as lower deep WM FA compared to participants without mTBI. Interestingly, more severe post-concussive symptoms, slower processing speed, poorer executive functioning, and poorer functioning in work, family, and social life were significantly associated with lower GM/WM boundary FA but not with deep WM FA. There were no group differences in cortical thickness. These findings suggest that microstructural alterations at the GM/WM boundary may be a sensitive marker of adverse effects of long-term outcomes following mTBI.

### 4.1. WM and GM Alterations Following mTBI

The group of participants with mTBI showed lower WM FA, compared to participants without mTBI. This is in line with some previous studies that report widespread lower WM FA in individuals with a history of mTBI [15]. When acceleration and rotational forces act on the skull, the strain is transferred to the brain’s tissue and may result in the tearing or shearing of axons. This shearing of axons has previously been associated with lower FA [46]. Previous research further suggest that lower FA may reflect gliosis, demyelination, and/or inflammation months to years post-injury [47].

In the current study, we also report diffusion alterations at the GM/WM boundary many years following mTBI, suggesting that shear strain at the GM/WM boundary may result in long-lasting pathology. Of interest here, a study using a computer simulation of head impact predicted that strain and strain rate are highest at the GM/WM boundary [10]. This is likely due to differences in tissue viscoelastic properties of WM compared to GM, which may increase the likelihood of shearing of GM against WM [8]. The GM/WM boundary may thus be particularly vulnerable to shear strain-induced axonal injury and subsequently also to TAI-related pathologies [10]. 

### 4.2. Association between GM/WM Boundary Diffusion and Long-Term Outcome Following mTBI

A large percentage (46.6%) of the mTBI group exhibited post-concussive symptoms years after injury (the average time since injury was 7.57 years in this sample), which is consistent with the literature [5]. Further, more severe post-concussive symptoms were associated with lower whole-brain and lobe-specific GM/WM boundary FA. This association was still significant when adjusting for common confounders of brain structure (i.e., age, gender, PTSD, depressive symptoms, and alcohol use) suggesting that WM alterations at the GM/WM boundary may be sensitive to adverse long-term outcomes following mTBI. Of further note, we observed lower GM/WM boundary FA values in the mTBI group, particularly in older participants with greater psychiatry symptom burden, potentially suggesting additive effects. 

In previous studies, lower deep WM FA post-injury has been associated with neuroinflammation, characterized by the activation of microglia, astrocytes, and the release of inflammatory cytokines, which exacerbates myelin loss, and impairs neurogenesis [47]. Altered microstructure at the GM/WM boundary may thus affect communication between brain regions that belong to functional connectivity networks [48], which have been associated with post-concussive symptoms [49]. Moreover, we hypothesize that degeneration or demyelination of axons at the GM/WM boundary may be associated with disruptions in communication between cortical and subcortical structures. Indeed, mTBI has been linked to altered functional connectivity within large-scale brain networks, such as the default mode network. Particularly intriguing, altered functional network activity has previously been observed in individuals who exhibit post-concussive symptoms [50]. 

In addition to an association between post-concussive symptoms and lower GM/WM boundary FA, we report an association between poorer functioning in work, family, and social life, and lower whole-brain, frontal, parietal, temporal, and occipital GM/WM boundary FA and deep WM FA. Long-term functional impairment is common after mTBI, and our results are consistent with findings that associate functional impairment with mTBI-related WM alterations [51]. Importantly, results from this study extend the current literature by suggesting that FA at the GM/WM boundary is a sensitive marker of functional impairment following mTBI.

Finally, lower whole-brain, frontal, and temporal GM/WM boundary FA were significantly correlated with slower processing speed, while lower parietal lobe GM/WM boundary FA was associated with poorer executive functioning. In this regard, mTBI has consistently been linked to reduced processing speed and impaired executive functioning [52]. In addition, effective communication across several brain areas is a prerequisite for both processing speed and executive functioning tasks [53]. The prefrontal cortex is particularly important for executive functioning, and damage to this area may result in impaired cognitive functioning that includes mental flexibility, task switching, attention, and decision making [54]. Indeed, the central executive network, a large-scale brain network that encompasses the dorsolateral prefrontal cortex and posterior parietal cortex [55], is commonly disrupted in chronic mTBI patients [50]. We speculate that by aggravating disrupted signal transmission across these cortical regions, mTBI-related injury at the GM/WM boundary may contribute to poor cognitive outcomes [56]. 

These findings, taken together, suggest that microstructural alterations at the GM/WM boundary may be sensitive markers of adverse long-term outcomes including processing speed, executive functioning, and functioning in work, family, and social life, following mTBI.

### 4.3. Limitations and Future Directions

We acknowledge several limitations in the current study. First, we analyzed cross-sectional data, precluding the inference of conclusions based on causal relationships between the study variables. Longitudinal studies are thus needed to evaluate changes in diffusion properties at the GM/WM boundary over time. Ideally, a large sample should be followed over time (i.e., pre-injury, within 24 h of injury, acutely, sub-acutely, and in the chronic stages post-injury). Further, for a comparison group, individuals with orthopedic injuries may be considered in addition to a group of uninjured individuals to mitigate bias. In addition, various imaging modalities should be correlated with neuropsychological outcomes that can be contrasted against each other to identify biomarkers of mTBI sensitive to adverse long-term outcomes. Second, the mTBI assessment was based on self-report, without available medical records or other information on injury mechanism, previous mTBI, and premorbid estimates of symptoms for validation. Third, gender, not biological sex, was assessed via the study’s demographic questionnaire. Unfortunately, we lack data on how many participants identified as a gender other than their biological sex. Fourth, while participants were recruited as part of the Department of Defense INTRuST Clinical Consortium, information about military status was not available, meaning we were unable to identify a participant as a civilian or a deployed combat veteran. This is important to know since causes of military mTBI have been shown to differ from causes of civilian mTBI, given that military blast-related injuries affect brain structure and function more profoundly than blunt injuries [57]. The mechanism of injury may also likely be relevant to the GM/WM boundaries that are subjected to the most strain [10]. 

Last, we investigated whole-brain GM, WM, and whole-brain and lobe-specific GM/WM boundary alterations, rather than focusing on individual cortical areas or specific WM tracts. Given the lack of research on mTBI-related alterations at the GM/WM boundary, we chose this approach to provide an initial broad overview of GM/WM boundary alterations and associated clinical burden. Moreover, delineating the GM/WM boundary and transferring low-resolution and echo planar imaging (EPI) distorted diffusion data onto the anatomical space is particularly challenging in small regions of interest compared to the entire brain. Future studies will likely benefit from employing a combination of structural and functional imaging to identify network hubs and more specific brain regions and their connections, which may be vulnerable to GM/WM boundary injury and associated post-concussive burdens. 

## 5. Conclusions

We report microstructure alterations at the GM/WM boundary in a group of individuals with a history of mTBI compared to a comparison group. Moreover, in the mTBI group, lower GM/WM boundary FA is associated with post-concussive symptom severity and poor clinical and cognitive functioning. We conclude that microstructural alterations at the GM/WM boundary may serve as a sensitive marker of mTBI-related adverse long-term outcomes following mTBI.

## Figures and Tables

**Table 1:** Sample Characteristics.

		Total Sample		mTBI		No mTBI			
								**ANCOVA**	
***Demographics***		*n*	mean ± SD	*n*	mean ± SD	*n*	mean ± SD	*F*(df)	*p*
Age		278	36.27 ± 12.71	147	36.56 ± 11.97	131	34.62 ± 12.88	1.70(1, 276)	0.193
Years between injury and scan		-	-	101	7.57 ± 9.54	-	-	-	-
								**Fisher’s exact test**	
		**%**		**%**		**%**		χ^2^	*p*
Gender (male/female)		54.3/45.7		65.3/34.7		41.9/58.1		15.31	<0.001
Race	Native	0.7		1.4		-		13.74	0.193
	Asian	3.2		1.4		5.3			
	Pacific	0.4		-		0.8			
	African American	13.7		10.2		17.6			
	White	76.3		82.9		68.7			
	Unknown	5.7		4.1		7.6			
								**ANCOVA**	
***Imaging***		*n*	mean ± SD	*n*	mean ± SD	*n*	mean ± SD	*F*(df)	*p (pFDR)*
Whole-brain GM/WM Boundary FA		278	0.27 ± 0.02	147	0.27 ± 0.02	131	0.28 ± 0.01	23.16(1, 271)	**<0.001 (0.001)**
Frontal lobe GM/WM Boundary FA		278	0.28 ± 0.02	147	0.28 ± 0.02	131	0.29 ± 0.02	18.62(1, 271)	**<0.001 (0.001)**
Parietal lobe GM/WM Boundary FA		278	0.27 ± 0.04	147	0.26 ± 0.05	131	0.28 ± 0.03	8.88(1, 271)	**0.003 (0.004)**
Temporal lobe GM/WM Boundary FA		278	0.28 ± 0.02	147	0.27 ± 0.02	131	0.28 ± 0.01	27.78(1, 271)	**<0.001 (0.001)**
Occipital lobe GM/WM Boundary FA		278	0.25 ± 0.02	147	0.25 ± 0.02	131	0.26 ± 0.02	6.65(1, 271)	**0.010 (0.011)**
Deep white matter FA		278	0.58 ± 0.02	147	0.57 ± 0.02	131	0.59 ± 0.02	25.21(1, 271)	**<0.001 (0.001)**
Whole-brain cortical thickness		278	2.37 ± 0.09	147	2.35 ± 0.08	131	2.37 ± 0.10	0.039(1, 271)	0.843 (0.843)
***Psychiatric Symptoms***								*F*(df)	*p*
PCL-C		278	30.40 ± 17.58	147	36.66 ± 18.50	131	23.30 ± 13.38	35.35(1, 274)	**<0.001**
PHQ-9		278	4.40 ± 5.67	147	6.61 ± 5.99	131	1.92 ± 4.06	47.72(1, 274)	**<0.001**
***Alcohol Use***									
AUDIT-10		278	3.73 ± 5.50	147	4.67 ± 6.40	131	2.67 ± 4.03	6.63(1, 274)	**0.011**
***Post-Concussive Symptoms***									
RPQ13		168	13.25 ± 15.04	139	17.35 ± 14.79	29	3.76 ± 11.23	21.83(1, 166)	**<0.001**
***Functional Impairment***									
SDS		275	6.36 ± 9.07	147	10.44 ± 9.70	128	1.67 ± 5.28	74.47(1, 271)	**<0.001**
***Cognitive Functioning***								*F*(df)	*p*
TMT-A time (seconds)		275	24.92 ± 10.21	145	26.58 ± 10.78	130	23.06 ± 9.24	6.10(1,271)	**0.014**
TMT-B time (seconds)		275	55.65 ± 27.52	145	58.19 ± 27.52	130	52.84 ± 26.83	0.89(1,271)	0.348

*Note.* SD, Standard deviation; PCL-C, PTSD Checklist Civilian; PHQ-9, Patient Health Questionnaire; SDS, Sheehan Disability Scale; AUDIT, Alcohol Use Disorder Identification Test, RPQ13, Rivermead Post-Concussion Questionnaires 13; All ANCOVAs were corrected for age and gender. Imaging parameters were corrected for age, gender, PCL-C, PHQ-9, and AUDIT-10. Significant *p*-values are marked in bold font.

**Table 2:** Acquisition parameters for MRI.

Sequence	Parameter	SIEMENS	PHILIPS	GE
DTI				
	Orientation	axial	axial	axial
	Phase Encoding Direction	a/p	p/a	l/r
	FOV (in mm)	256	256	256
	Bandwidth (in kHz or Hz/Px)	1396	1271	250
	Number of Directions	87	64	86
	b-value	900	900	900
	Number of b0	0	7	1
	Resolution Matrix	128 × 128	128 × 128	128 × 128
	Voxel Size (in mm^3^)	2 × 2 × 2	2 × 2 × 2	2 × 2 × 2
	Number of Slices	73	73	73
	Acquisition Time (in min)	14:08	14:21	14:40
T1w				
	Sequence details	MP-RAGE	T1W_3D_TFE SENSE	SPGR-BRAVO
	Orientation	Sagittal	Sagittal	Sagittal
	Flip Angle (in degrees)	7	7	10
	FOV (in mm)	256	256	256
	Bandwidth (in kHz)	25.6	24.5	25.0
	TE (in ms)	3.3	3.5	3.7
	TR (in ms)	2530	7600	9150
	Inversion Time	1100	1100	600
	Resolution Matrix	256 × 256	256 × 256	256 × 256
	Voxel Size (in mm^3^)	1 × 1 × 1	1 × 1 × 1	1 × 1 × 1
	Number of Slices	176	176	176
	Acquisition Time (in min)	6:03	5:13	5:15

*Note*. Multi-site study; MRI data acquisition on Tim Trio, Siemens Healthineers, Erlangen, Germany; GE 750, GE Healthcare, Chicago, IL, USA; Achieva, Philips Healthcare, Best, The Netherlands. FOV, Field of view; TE, Echo time; TR, Repetition time.

## Data Availability

The data presented in this study is available on request from the corresponding author.
